# Sulfoxaflor influences the biochemical and histological changes on honeybees (*Apis mellifera* L.)

**DOI:** 10.1007/s10646-023-02677-0

**Published:** 2023-06-17

**Authors:** El-Desoky S. Ibrahim, Asmaa E. Abd Alla, Mohamed S. El-Masarawy, Rasha A. Salem, Nancy N. Hassan, Moataz A. M. Moustafa

**Affiliations:** 1 Department of Economic Entomology and Pesticides, Faculty of Agriculture, Cairo University, 12613 Giza, Egypt; 2 Bee Res. Dep., Inst. Plant Protec. Res., Agric. Res. Center, Giza, Egypt

**Keywords:** *Apis mellifera*, Sulfoxaflor, Toxicity, GST, MFO, Histological effects

## Abstract

Pesticide application can have an adverse effect on pollinator honey bees, *Apis mellifera* L., ranging from mortality to sublethal effects. Therefore, it is necessary to understand any potential effects of pesticides. The present study reports the acute toxicity and adverse effects of sulfoxaflor insecticide on the biochemical activity and histological changes on *A. mellifera*. The results showed that after 48 h post-treatment, the LD_25_ and LD_50_ values were 0.078 and 0.162 µg/bee, respectively, of sulfoxaflor on *A. mellifera*. The detoxification enzyme activity shows an increase of glutathione-S-transferase (GST) enzyme on *A. mellifera* in response to sulfoxaflor at LD_50_ value. Conversely, no significant differences were found in mixed-function oxidation (MFO) activity. In addition, after 4 h of sulfoxaflor exposure, the brains of treated bees showed nuclear pyknosis and degeneration in some cells, which evolved to mushroom shaped tissue losses, mainly neurons replaced by vacuoles after 48 h. There was a slight effect on secretory vesicles in the hypopharyngeal gland after 4 h of exposure. After 48 h, the vacuolar cytoplasm and basophilic pyknotic nuclei were lost in the atrophied acini. After exposure to sulfoxaflor, the midgut of *A. mellifera* workers showed histological changes in epithelial cells. These findings of the present study showed that sulfoxaflor could have an adverse effect on *A. mellifera*.

## Introduction

Over 90% of flowering plant species in hot and humid environments require pollination to produce healthy fruit (Ollerton et al. 2011). Animal pollination is essential for reproduction of most flowering plants (Kremen et al. 2007). Loss of insect pollinators is a menace for global food security. Honeybees are globally considered essential pollinators in crops, fruits-bearing plants and wild species (Winfree et al. 2008). With remarkable success, bees pollinate 71 common crops from hundreds of plant species that make up 90% of the world’s food supply (Morse and Calderone 2000; Gallai et al. 2009; Artz et al. 2011). However, recently some world regions have been suffering from an increase in losses from their managed honey bee colonies. Colony Collapse Disorder (CCD) was first reported in 2006 in the USA (Neumann and Carreck 2010). The interaction between environmental stress factors, particularly exposure to pesticides and pathogens, is believed to be the main potential cause of colony collapse. It is difficult to define the main reasons for loss of colonies due to the varied social behavior of bees. They are exposed to daily human activities and other environmental factors. While numerous factors lead to losses, new reports have shown some of these factors include bee-keeping practices, pests, diseases, pesticide use, agricultural practices, and climate change (Hristov et al. 2021). Previous investigation has reported that exposure to insecticides affects colony stability (Henry et al. 2012) and homing capacity of honey bees (Tosi et al. 2017; Fulton et al. 2019), as well as memory neuronal inactivation in the mushroom body, olfactory learning, bumble bee colony growth, and queen production (Whitehorn et al. 2012). Pesticides can disrupt physiological processes unrelated to the intended modes of action (Chakrabarti et al. 2015). For example, they can cause oxidative stress and apoptosis (Gregorc et al. 2018**)** in bees and can exhibit smaller and irregularly shaped hypopharyngeal glands in *A. mellifera* (Menail et al. 2020). Neonicotinoids are the most studied pesticides in terms of side effects on pollinators with emphasis on their impacts on different bee species (Tsvetkov et al. 2017). Sulfoxaflor, the first commercial insecticide of the sulfoximines, is a systemic insecticide that translocates via treated crop plants and may contaminate pollen and nectar (Giorio et al. 2021; EFSA 2019). Sulfoxaflor acts as antagonist of the nicotinic acetylcholine receptor (nAChRs). The binding affinity of sulfoxaflor to the receptor makes it a distinctive insecticide compared to other nAChRs antagonists, which could be somewhat less toxic than other neonicotinoids such as imidaclocloprid and clothianidin (Azpiazu et al. 2021). As it is responsible for the digestion and absorption of ingested food, the insect’s midgut is a vital organ for toxicity studies. Additionally, chemical ingestion can affect other non-target insect organs as insecticides enter the midgut barrier and get distributed in the hemolymph (Catae et al. 2018) and damage mushroom bodies in the brain (de Morais et al. 2018). Sulfoxaflor is an effective insecticide against several sucking insect pests which are resistant to other insecticide classes including resistant species to the neonicotinoids. Due to its lack of cross-resistance, sulfoxaflor is a poor substrate for the metabolic enzymes endowing resistance to other classes of insecticides (Sparks et al. 2013).

Chronic exposure to Sulfoxaflor at field-recommended concentrations has been shown to diminish egg-laying and impair reproductive success in bumblebees (Siviter et al. 2018, 2020). In contrast, no effect was found on learning and memory of bumblebees after acute exposure to sulfoxaflor (Siviter et al. 2019) or the escape response of locusts (Parkinson et al. 2020). Exposure of *A. mellifera* colonies to Sulfoxaflor in a flight enclosure caused acute toxicity but did not otherwise impact flight activity or long-term colony development (Cheng et al. 2018). Sulfoxaflor has recently been shown to increase oxidative stress and induce apoptosis in *A. mellifera* (Chakrabarti et al. 2020). So far, it has been shown that exposure of honey bees to sublethal doses of insecticides could affect motor activity, feeding, development, reproductive system, and enzyme (antioxidant and detoxification) mechanisms (Murawska et al. 2021). In contrast, sulfoxaflor has a low synergistic effect in bee species after treatment of bees with the LD_50_ value of sulfoxaflor alone or in combination with the fungicide fluxapyroxad (Azpiazu et al. 2021). Thus, in a semi-field study, no significant effects of sulfoxaflor and the fungicide azoxystrobin were found on bees (Tamburini et al. 2021). While, both forms of chlorntraniliprole (technical-grade and formulation product) had a toxic effect after 4 or 72 h post-treatments (Williams et al. 2020). On the other hand, few studies have demonstrated the detoxification enzymes to conventional insecticides in honeybees (Papadopoulos et al. 2004; Johnson et al. 2006).

Generally, the lethal and sublethal exposure to chemical or bio-insecticides may lead to changes in the biochemical and physiological characteristics of insects (Awad et al. 2022; Moustafa et al. 2022). Exposure of honey bees to insecticides may effect their motor activity, feeding, development, reproductive system and enzyme activities. Therefore, the aim of our work was to evaluate the effect of LD_25_ and LD_50_ values of sulfoxaflor on honey bee. Our present work aims to assess the toxicity and the adverse effects of the insecticide sulfoxaflor on the activity of detoxification enzymes (Glutathione-*S*-Transferase and Mixed-Function Oxidases). In addition, we investigated the damage caused by sulfoxaflor in histopathological alteration for each brain, midgut and the hypopharyngeal gland of *Apis mellifera*.

## Materials and methods

### *Apis mellifera* samples

Adult worker bees (≥21days) were collected from adequately fed, healthy, disease-free, and queen-right colonies with known history and physiological status at the apiary yard of the Faculty of Agriculture, Cairo University, Egypt. The collected bees were reared on sucrose solution in water (50% w/v) at a temperature of 25 ± 2 °C with a relative humidity of 60–70%.

### Bees bioassay

Bees were assembled individually without touching with hands directly to the 15 mL falcon tube (this will make bees move normally) with ventilation holes. Non-anesthetization methods, including CO2, ether and low temperature, were used to reduce mortality. Bees were collected in the early morning directly from the opening of a tunnel, and we starved them for 2 h in the incubator before the test. Five doses were prepared in a geometric series of sulfoxaflor including; 0.039, 0.078, 0.156, 0.321, and 0.625 mg/L to determine the LD values. Ten µL of each sugary concentration solution was applied to the lid of the falcon tube. Then the tube was inverted so that it was in the bottom and turned it upside down to allow the bee to feed on. We calculated consumption by weighing the empty tube cover for each tube and then added 10 µL droplets of the pesticide in sugary solution to the cover using an adjustable micropipette (2–20 microliter). Three replicate groups of ten separated bees were conducted. For the control treatment, individual bees were fed sucrose only. After feeding the bees on the treated or untreated diet for 4 h, the live bees were transferred onto a new falcon tube attached with a 1.5 mL of Eppendorf tube that stands vertically with sucrose solution only. The mortality percentage (%) of bees was measured and recorded after 24 and 48 h post-treatment. This experiment was conducted twice.

#### Biochemical analysis

##### Sample preparation

Two adult honey bees were collected from each replicate cage (3 replicates) after treating the honey bees with LD_25_ and LD_50_ values of sulfoxaflor as mentioned above. Each sample was homogenized in phosphate buffer saline pH 7.4 according to according to Chakrabarti et al. (2020) using a TT-30K digital handheld homogenizer (Hercuvan Lab System, Malaysia) at 8000 rpm for two cycles of 30 s /cycle. The homogenates were centrifuged at 10,000 rpm for 5 min at 4 °C. The supernatants were kept at −20 °C until they were used.

##### Glutathione- S-Transferase (GST) assay

GST activity was determined according to Habig et al. (1974) and Moustafa et al. (2021a). The reaction solution was composed of 10 µL of homogenate sample as enzyme stock solution, 25 µL 30 mM CDNB, and 25 µL 50 mM GSH. The GST activity was measured at 340 nm at 25 °C for 3 min using a spectrophotometer (Jenway-7205 UV/Vis, Staffordshire, UK).

##### Mixed-Function Oxidases (MFO)

MFO activity was tested according to Hansen and Hodgson (1971) and Moustafa et al. (2021b). First, we incubated 100 µL of 2 mM p-nitro anisole with 90 µL of homogenate sample for 2 min at 27 °C, and we added 10 µL of 9.6 mM NADPH to initiate the reaction. Then, the activity of MFO was measured at 405 nm for 15 min using molecular devices of microplate reader (Clindiag-MR-96, ISO09001:2008, Steenberg, Belgium). Finally, we used the standard curve of p-nitrophenol to calculate the MFO activity.

### Histopathological studies

Seven adult honeybees were used for each treatment; each worker bee was anesthetized by cold exposure (4 °C) and carefully dissected (Dade 1977) with a fine pointed watchmaker forceps (Dumont, No: 5) and dissection scissors (Hammacher, Solingen) under a stereomicroscope (Euromex). First, we pinned the live bees onto a glass petri dish filled with paraffin by insect pins to prevent them from moving. Then, the cuticle was removed by cutting both sides of the abdomen to expose the internal organs (midgut). Finally, the head was dissected to remove the brain and hypopharyngeal gland.

Autopsy samples were taken from the brain, hypopharyngeal gland, and main gut of bees in different groups fixed in 10% formol saline for twenty-four hours. We used tap water to wash, then serial dilutions of alcohol (methyl, ethyl, and absolute ethyl) for dehydration. We cleared specimens in xylene and embedded them in paraffin at 56 °C in a hot air oven for 24 h. We prepared paraffin bees wax tissue blocks for sectioning at 4-micron thickness by slide microtome. Then, the tissue sections were collected on glass slides, deparaffinized and stained with hematoxylin-eosin for examination through the electric light microscope (Banchroft et al. 1996).

### Data analyses

The statistical analysis program LDP line was used to determine the LD values for sulfoxaflor insecticide (with 95% confidence limits). We performed a one-way ANOVA for the enzymatic activity using SAS software (SAS 2001). We separated the mean values with the Duncan’s multiple range test.

## Results

### Effects of sulfoxaflor on *A. mellifera*

After 48 h post-treatment, the LD_25_ and LD_50_ values were 0.0785 and 0.1623 µg/bee, respectively with 95% confidence limit of 0.0511–0.1045 and 0.1242–0.2155, respectively (Table 1) of sulfoxaflor on *A. mellifera*. In addition, exposures to sulfoxaflor caused changes in the level of the enzyme activity of GST (Table 2). The results indicated that sulfoxaflor caused a significant increase in GST activity after 12, 24 and 48 h of post-treatment [F = 33.72, 17.51 and 110.23, *P* = <0.0001, 0.0008 and <0.0001] at the LD_50_ (2.3, 1.58 and 1.29-fold, respectively) compared to the control treatment. In contrast, sulfoxaflor at LD_25_ caused a significant decrease in the GST activity compared to the control (Table 2). The activity of MFO showed no significant differences after treating the *A. mellifera* with LD_25_ and LD_50_ of sulfoxaflor [F = 13.52, 0.23, 1.71 and 1.24, *P* = 0.001, 0.793, 0.234 and 0.333] compared with the control group (Table 3).

**Table 1:** Toxicity of sulfoxaflor on *A. mellefera*

Insecticide	Values of LDs			Slope±SE
	LD_25_ (µg/ml) (95% Confidence Limit)	LD_50_ (µg/ml) (95% Confidence Limit)	LD_90_ (µg/ml) (95% Confidence Limit)	
Sulfoxaflor	0.0785 0.0511–0.1045	0.1623 0.1242–0.2155	0.6443 0.4277–1.3449	2.140 ± 0.344

**Table 2:** Mean (±SE) of GST enzyme activity of *A. mellefera* after treated with LD_25_ and LD_50_ values of sulfoxaflor

Treatments	GST (μmole/bee/mg of protein)			
	4 h	12 h	24 h	48 h
Control	0.372^a^ ± 0.04	0.336^b^ ± 0.01	0.378^b^ ± 0.08	0.334^b^ ± 0.01
LD_25_	0.253^ab^ ± 0.03	0.133^c^ ± 0.02	0.151^c^ ± 0.01	0.123^c^ ± 0.01
LD_50_	0.186^b^ ± 0.04	0.787^a^ ± 0.09	0.601^a^ ± 0.03	0.433^a^ ± 0.02
F	4.82	33.72	17.51	110.23
*P*-value	0.0377	<0.0001	0.0008	<0.0001

Means within a column followed by different letters are significantly different (*p* < 0.05).

**Table 3:** Mean (±SE) of MFO enzyme activity of *A. mellefera* after treated with LD_25_ and LD_50_ values of sulfoxaflor

Treatments	MFO (µmole/bee/mg of protein)			
	4 h	12 h	24 h	48 h
Control	0.107^a^ ± 0.01	0.106^a^ ± 0.001	0.114^a^ ± 0.008	0.114^a^ ± 0.01
LD_25_	0.143^a^ ± 0.02	0.126^a^ ± 0.02	0.090^a^ ± 0.02	0.083^a^ ± 0.01
LD_50_	0.053^b^ ± 0.02	0.121^a^ ± 0.02	0.069^a^ ± 0.01	0.093^a^ ± 0.006
F	13.52	0.23	1.71	1.24
*P*-value	0.0019	0.7955	0.2340	0.3337

Means within a column followed by different letters are significantly different (*p* < 0.05).

### Histopathological changes of brain, hypopharyngeal glands and midgut of *A. mellifera*

#### Brain

**Fig. 1:**
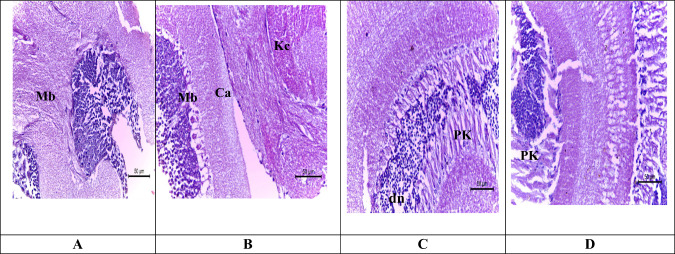
Light micrographs of the brain of *Apis mellifera L*. (H&E X160) **A** the control bees showing no histopathological alteration **B** Brain of treated bees administrated insecticide (sulfoxaflor) for 4 h. Showing nuclear pyknosis (pk) and degeneration in some few cells. **C** Brain of treated bees with administrated insecticide (sulfoxaflor) For 24 h. showing most of the neuronal cells have nuclear pyknosis and degeneration. (**D**) (x80) after 48 h. The mushroom shaped tissue showed loss of most neurons and replaced by vacuoles in magnification showing vacuolization replacing the damage neurons. **Nb:** mushroom body (**Mb**), calyx (**Ca)**, kenyon cells (**kc**), pyknosis (**pk**), damage neurons(**dn**)

We observed no histopathological alteration in the control of *A. mellifera* brain (Fig. 1A). The brain of *A. mellifera* L. manifests normal structure, Kenyon cells of the mushroom bodies were apparent, well-developed spherical nuclei and clear nucleoli. After 4 h the brain of treated bees with insecticide (sulfoxaflor) showed nuclear pyknosis and degeneration in some cells (Fig. 1B). The histopathology brain of bees treated with sulfoxaflor shows that most neuronal cells have nuclear pyknosis and degeneration for 24 h (Fig. 1C). After 48 h, the mushroom-shaped tissue showed loss of most neurons and was replaced by vacuoles in magnification.

#### Hypopharyngeal gland

**Fig. 2:**
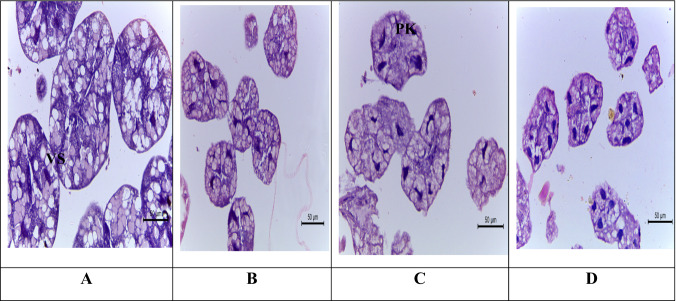
Light micrographs of the hypopharyngeal gland of *Apis mellifera* L. (H&E X160). **A** the control bees showing cytoplasm of the secretory cell seen contain a variable number of secretory vesicles that are almost unstained. **B** hypopharyngeal gland of treated bees with administrated insecticide (sulfoxaflor) for 4 h, showing slightly effect in secretory vesicles (vs). **C** hypopharyngeal gland of treated bees with administrated insecticide (sulfoxaflor) for 24 h, showing atrophy with nuclear pyknosis (pk) in most of the cells showing loss of cytoplasmic fat vacuoles. (**D**) (X80) after 48 h. There were loss of the vacuolar cytoplasm and basophilic pyknotic nuclei in the atrophied acini

The hypopharyngeal gland of *A. mellifera* L. contains secretory cells, the cytoplasm of which contains a variable number of secretory vesicles that appear almost as unstained as the control (Fig. 2A). There was a slight effect in secretory vesicles of treated bees with sulfoxaflor after 4 h (Fig. 2B). After 24 h, the treated bees showed atrophy with nuclear pyknosis in most cells, showing a loss of cytoplasmic fat vacuoles (Fig. 2C). After 48 h, the vacuolar cytoplasm and basophilic pyknotic nuclei were lost in the atrophied acini.

#### Midgut

**Fig. 3:**
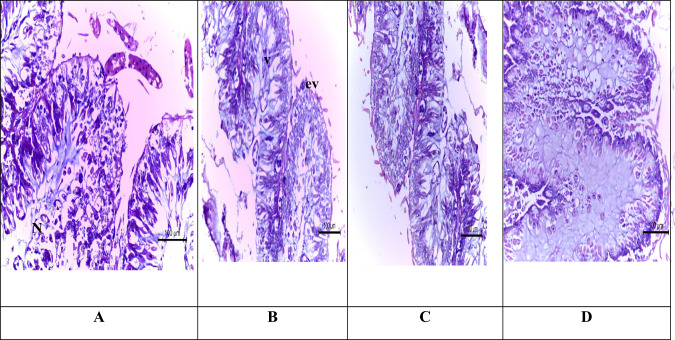
Light micrographs of the midgut of *Apis mellifera* L. (H&E X160) **A** the control bees showing single layered epithelium with columnar cells containing spherical nucleus (N) and apical surface with peritrophic matrix layers (pm). **B** Midgut region of treated bees with administrated insecticide (sulfoxaflor) for 4 h. Showing vacuolar degeneration lumen (L) epithelium of villi (ev). **C** Midgut region of treated bees with Administrated Insecticide (sulfoxaflor) for 24 h. showing necrobiotic change was detected in the lining epithelial cells. (**D**) (x80) after 48 h. The mucosal lining epithelium showed necrosis with lose of the histological structure and replaced by pigmented material. Other’s areas of the mucosa had vacuolar degenerative changes

The midgut epithelial cells of *A. mellifera* workers (control) show normal nuclei, and the cytoplasm was densely homogeneous (Fig. 3A). After 4 h exposure to sulfoxaflor, the midgut epithelial cells of *A. mellifera* workers showed epithelium villi, many cytoplasmic vacuoles, and degenerated vacuolar lumen. The midgut region of bees treated with sulfoxaflor for 24 h showed the necrobiotic change in the lining epithelial cells. After 48 h, the mucosal lining epithelium showed necrosis with loss of the histological structure and was replaced by pigmented material. Other areas of the mucosa had degenerative vacuolar changes.

## Discussion

This study provides new insight into sulfoxaflor’s lethal and sublethal effects on honeybees. To understand this insecticide’s negative effects, both mortality and biochemical parameters on *A. mellifera* were measured. Results confirmed that sulfoxaflor is very toxic (LD_50_ = 0.078 µg/mL) to honeybees, which have fewer detoxification genes than insect pests, thus making honeybees more susceptible to pesticide exposure (Sadd et al. 2015). Other studies have classified sulfoxaflor toxicity on honeybees with oral and contact LD_50_ values of 0.05 and 0.13 µg/mL a.i/bee, respectively (Cheng et al. 2018), while its LC_50_ value was 1.72 mg/L after 96 h post-treatment (Li et al. 2021). In parallel, in the Pesticide Properties Data Base, the contact LD_50_ of sulfoxaflor was 0.379 μg/bee and for acute LD_50_ was 0.146 μg/bee for honeybees (Li et al. 2021).

The biochemical and physiological effects of insecticides can render individual honeybees unable to perform their mission smoothly, thus affecting the colony’s performance (Chakrabarti et al. 2020). GST and MFO are important metabolic enzymes and are essential elements in developing insecticide detoxication on insects (Bird et al. 2022). In the current study, GST exhibited higher activity in *A. mellifera* to sulfoxaflor. In addition, the activity of mixed-function oxidase did not significantly increase in bees. These results agree with Hu et al. (2014) and Zhang et al. (2016) who found an overexpression of GST related to resistance to diamide insecticides. There is also a positive correlation between the toxicity of Lambda-cyhalothrin and the activity of GST enzyme (Zhu et al. 2017), while no effect on esterase and GST activity was observed after spraying *A. mellifera* with sulfoxaflor (Zhu et al. 2017).

Thus, the exposure of honey bee workers to sulfoxaflor for 48 h caused a negative effect on the mushroom bodies. This damage may affect walking behavior (Martin et al. 1998; Helfrich-Förster et al. 2002), reduce memory ability and learning (Peng and Yang 2016) and influence the sensory organs in the head (Hansson and Anton 2000; Paulk et al. 2009; de Morais et al. 2018). As shown above, there was a slight effect in secretory vesicles after 4 h from exposure to sulfoxaflor, while after 24 h the effect has increased to atrophy with nuclear pyknosis in most of the cells showing loss of cytoplasmic fat vacuoles and after 48 h increased in basophilic pyknotic nuclei in the atrophied acini. This corresponds with the findings of several authors who confirmed the same histological effects after exposure to insecticides. When honey bees are exposed to insecticides, the cytoplasm degenerates and the nuclei of the secretory vesicles of the hypopharyngeal gland undergo pyknosis, which ultimately results in vacuoles and heterogeneous secretory vesicle content (Halberstadt 1980; Silva de Moraes and Bowen 2000).

Minor targets in insects may be affected. Although the insecticide’s major target is (nAChR) in insect nervous systems (Tomizawa and Casida 2003; Tan et al. 2007) and the organs concerned the metabolism. The ultrastructure changes were revealed through ultrastructural study. Structures harmed by being exposed to sulfoxaflor were given to bees. This finding corresponds with Oliveira et al. (2014), who showed that morphological ultrastructure in the midguts of adults and larvae of *A. mellifera* are exposed to insecticides. A study on the midgut of Africanized honeybee with a dose of 0.428 ng/mL of thiamethoxam per day reduced the number of regenerative cells in the epithelium and incited cytoplasmic vacuolization. The midgut is the part of the digestive tract responsible for most food processing and absorption, and it is known as the functional stomach (Cruz-Landim 2009). Because the pesticide was taken orally, the midgut was one of the first organs to be exposed to the lethal dose, and it suffered immediate consequences at the start of the exposure. In addition, our results correspond with Abd Alla and El-Wassef (2019) who reported that the neonicotinoids insecticides were lethal for worker honeybees and revealed changes that happened in the midgut. The nuclei were abnormal, small-size and deep-blue color (nucleus pyknosis), most of the lining mucosal epithelium showed vacuolar degeneration in the cytoplasm after being treated with a recommended dose of imidacloprid.

## Conclusions

This study elucidated the adverse effects of sulfoxaflor insecticide on *A. mellifera*. Sulfoxaflor caused significant effects on mortality after 48 h post-treatment. Conversely, treated bees show significant differences in GST enzyme compared with the control group. The above findings of the present study show that sulfoxaflor has an adverse effect on tissue structure in the different organs (brain, hypopharyngeal gland and midgut) of the worker honeybee *A. mellifera* L, which eventually leads to death.
